# POIFormer: A Transformer-Based Framework for Accurate Point-of-Interest Attribution

**DOI:** 10.1145/3748636.3762775

**Published:** 2025-12-12

**Authors:** Nripsuta Ani Saxena, Shang-Ling Hsu, Mehul Shetty, Omar Alkhadra, Cyrus Shahabi, Abigail L. Horn

**Affiliations:** University of Southern California, Los Angeles, California, USA; University of Southern California, Los Angeles, California, USA; University of Southern California, Los Angeles, California, USA; University of Southern California, Los Angeles, California, USA; University of Southern California, Los Angeles, California, USA; University of Southern California, Los Angeles, California, USA

**Keywords:** point-of-interest attribution, mobility modeling, transformers

## Abstract

Accurately attributing user visits to specific Points of Interest (POIs) is a foundational task for mobility analytics, personalized services, marketing and urban planning. However, POI attribution remains challenging due to GPS inaccuracies, typically ranging from 2 to 20 meters in real-world settings, and the high spatial density of POIs in urban environments, where multiple venues can coexist within a small radius (e.g., over 50 POIs within a 100-meter radius in dense city centers). Relying on proximity is therefore often insufficient for determining which POI was actually visited. We introduce POIFormer, a novel Transformer-based framework for accurate and efficient POI attribution. Unlike prior approaches that rely on limited spatiotemporal, contextual, or behavioral features, POIFormer jointly models a rich set of signals, including spatial proximity, visit timing and duration, contextual features from POI semantics, and behavioral features from user mobility and aggregated crowd behavior patterns–using the Transformer’s self-attention mechanism to jointly model complex interactions across these dimensions. By leveraging the Transformer to model a user’s past and future visits (with the current visit masked) and incorporating crowd-level behavioral patterns through pre-computed kernel density estimates (KDEs), POIFormer enables accurate, efficient attribution in large, noisy mobility datasets. Its architecture supports generalization across diverse data sources and geographic contexts while avoiding reliance on hard-to-access or unavailable data layers, making it practical for real-world deployment. Extensive experiments on real-world mobility datasets demonstrate significant improvements over existing baselines, particularly in challenging real-world settings characterized by spatial noise and dense POI clustering.

## 1 Introduction

The widespread adoption of mobile devices and location-aware services has dramatically increased the availability of location-based data, and a growing body of research leverages mobility data for various downstream geo-spatial tasks, such as next-location prediction [13, 33, 46, 47] and detecting anomalies in movement patterns [50]. A critical first step in these modeling tasks is to contextualize raw GPS trajectories by associating them with semantically-informative user-visited places, or *points of interest (POIs)* – a process known as *POI attribution* [19, 51].

More specifically, POI attribution refers to the task of associating a user’s stay at raw GPS coordinates to a specific location, such as a store, restaurant, or public park. This mapping adds semantic context to otherwise opaque spatial data, enabling more interpretable and actionable insights into human behavior. For example, identifying that a user visited a coffee shop in the afternoon is significantly more informative than simply noting their presence at coordinates < latitude, longitude > at time t.

Moreover, effective POI attribution does not merely annotate location data–it enhances the *precision* of spatial understanding. In other words, it improves our ability to interpret and use spatial data accurately [38]. Distinguishing which specific establishment a person visited in a multi-store complex (such as a strip mall, for example) yields far more granular and useful behavioral insights than simply knowing they were near the area. This level of precision is vital across various applications, including location-based marketing [3, 6], urban planning [27, 38], and public health interventions such as pandemic hotspot detection [18]. Conversely, misattributing GPS data to the wrong POIs can risk contaminating downstream models to learn misleading or entirely spurious patterns, undermining their reliability and interpretability.

Even though accurate attribution is essential for downstream utility, POI attribution remains underexplored in the literature. This limited attention is likely due to the inherent challenges of the task. GPS signals are frequently noisy, and in densely populated areas where many POIs are located in close proximity, pinpointing the true destination is non-trivial. For instance, there are 753 retail businesses per square mile in downtown Los Angeles [37], creating significant spatial ambiguity. Ambiguity becomes unavoidable when the distance between adjacent POIs falls within the 5–15 meter error margin typical of the GPS capabilities of modern smartphones (Table 1). Alternative positioning methods, such as Wi-Fi or cellular network triangulation, offer broader coverage but at the cost of even lower precision.

**Figure 1:**
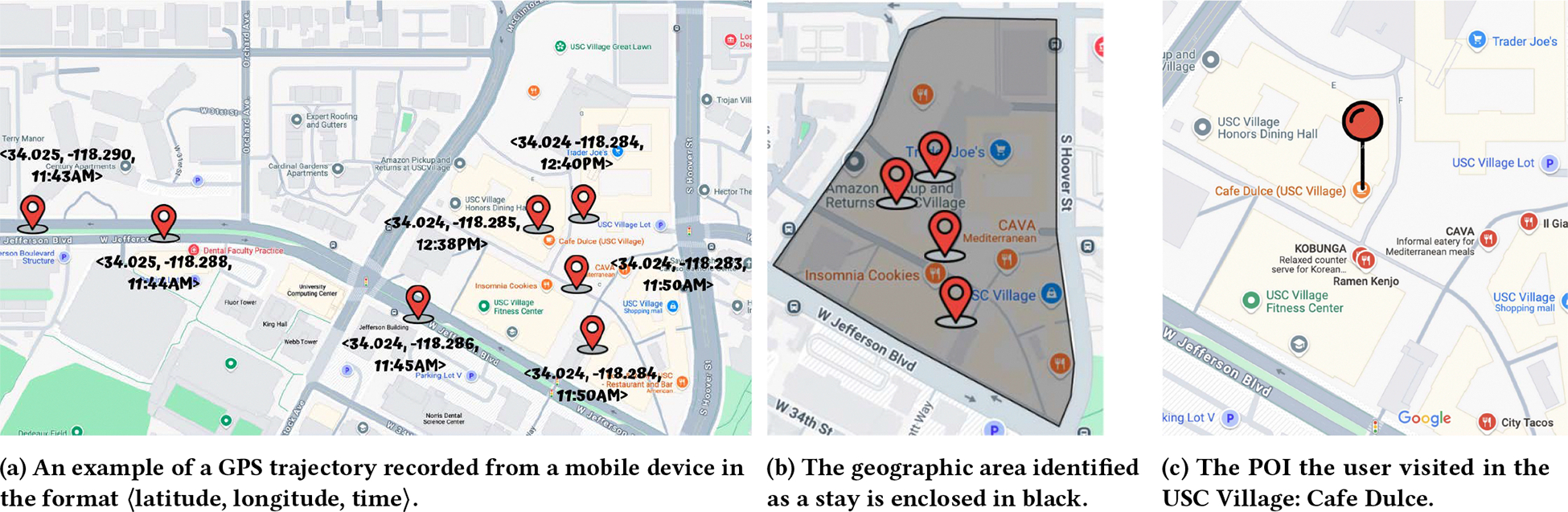
Illustration of key concepts in mobility analysis: Figure 1a showcases a GPS trace recorded from an individual’s mobile device over time. Figure 1b shows the identification of a stay point based on significant time spent in the area by the individual. Finally, Figure 1c shows the attribution of the stay to a specific POI (Cafe Dulce) within the stay point area.

Yet, despite this complexity, attribution is often reduced to a simple heuristic: assign each stay to the nearest POI [34]. While this approach is easy to implement, it fails to account for key real-world complexities, including GPS noise and spatial densities that can exceed precision bounds, or to leverage informative contextual cues such as time of day or visit duration. For example, consider a stay detected in USC Village, a university campus neighborhood in Los Angeles County. Even if GPS points cluster near Cafe Dulce (Figure 1), nearby venues–such as a dining hall or clothing store–fall well within the typical error margin of A-GPS or Wi-Fi or cellular positioning (Table 1). Such scenarios are common in dense urban areas, where POIs are tightly packed together. In these cases, proximity alone is insufficient for reliable attribution. Real-world settings such as this underscore the need for more sophisticated POI attribution models that account for spatial uncertainty and incorporate environmental context and historical user behavior to *predict* the POI visited.

Recent POI attribution methods have moved beyond simple spatial proximity, incorporating signals such as dwell time (i.e., the duration of the stay), user history, or POI semantic information–structured information describing the nature and function of a place (e.g., identifying a location as a cafe, a clinic, or a supermarket, along with attributes like opening hours, typical visitation patterns, or business category). For example, Nishida et al. [29] propose a hierarchical Bayesian model that leverages semantic information from POI categories–useful for making inferences into what types of POI a user is more likely to visit–along with individual-level behavioral signals. Suzuki et al. [41] develop a privacy-preserving approach that attributes visits based solely on a single individual’s mobility history, without aggregating across users. Finally, the current state-of-the-art, developed by the data company SafeGraph [35], enriches attribution with detailed spatial features like building footprint polygons and spatial hierarchy metadata that capture spatial relationships among locations. For instance, the hierarchical metadata can indicate that a store is located within a mall or that a food outlet is situated in a food court, providing valuable contextual information that supports more accurate POI attribution. Collectively, these methods demonstrate the growing recognition that POI attribution benefits from incorporating POI semantics alongside behavioral and contextual signals. However, despite this progress, most of these approaches face limitations including reliance on unrealistic assumptions, computationally intensive frameworks that limit scalability, and missed opportunities to leverage additional contextual signals. For example, Nishida et al. [29] assume that each POI belongs to a single category, which overlooks the multi-functional nature of many real-world locations, such as transit hubs that also serve as commercial centers. Moreover, while their model incorporates a POI’s visitation pattern as part of its semantic information, it does not explicitly leverage population-level mobility behaviors. As demonstrated in Section 4, these limitations collectively limit its accuracy. While the approach by Suzuki et al. [41] meets requirements of some privacy-preserving frameworks, their reliance on integer linear programming introduces prohibitive computational costs, limiting scalability and utility in non-restrictive settings. While the SafeGraph approach incorporates more detailed spatial contextual information, it omits information from behavioral signals such as individual visit history, population-level patterns, and personal preferences that can be leveraged for more accurate attribution. Furthermore, the comprehensive spatial data layers required by the SafeGraph approach are often unavailable or incomplete in many POI datasets and regions of the world, limiting its generalizability. While these methods demonstrate the growing recognition of the value of incorporating novel features such as POI semantics, none effectively leverage population-level behavioral mobility patterns that can enhance attribution accuracy in real-world environments.

To address these limitations, we propose POIFormer, a novel Transformer-based framework for POI attribution that jointly models a diverse set of signals, including spatial proximity, temporal features of the visit (arrival/departure and dwell time), POI semantics, user-specific mobility patterns, and population-level historical trends. A key innovation of POIFormer is its explicit incorporation of two dimensions of behavioral context: one capturing individual preferences, and another capturing crowd-level visit patterns. Individual preferences are modeled using a transformer that considers both past and future visits, with the location of the current (target) visit masked. This context enables the transformer to evaluate which nearby POI candidate is most likely given a user’s past and future visits, based on the time of day and duration of the stay of the target visit. Crowd-level historical visit patterns are modeled using the temporal popularity distributions of POIs, estimated via Kernel Density Estimation (KDE). These KDE models capture the joint distribution of location and time (e.g., hour of day) for visits within each POI category. This enables POIFormer to probabilistically downweight unlikely POIs–for example, reducing the likelihood of assigning a late-night visit to a coffee shop if historical data shows it is rarely visited at that hour. These KDEs are pre-computed per category facilitating efficient, scalable inference without sacrificing accuracy since they retain the full joint distribution of location and time while avoiding the need for computation at time of inference. Finally, POIFormer combines individual and crowd-level scores into a unified likelihood measure, selecting the most probable POI (or set of POI) among nearby candidates. Furthermore, unlike prior approaches [29, 35], POIFormer makes no restrictive assumptions about POI categories, and does not rely on detailed spatial data layers about POIs, thereby enhancing its applicability across diverse geographic and data-constrained contexts. Extensive experimental evaluation on publicly available datasets, one simulated and one derived from real-world mobility traces, demonstrate that POIFormer consistently outperforms existing baselines including the current state-of-the-art technique proposed by SafeGraph [35] by a substantial margin, particularly in top-3 and top-5 accuracy.

The paper is organized as follows. Section 2 reviews relevant prior work on POI attribution. Section 3 introduces our proposed method, POIFormer, detailing its architecture and components. Experimental results and evaluation are presented in Section 4. Finally, Section 5 concludes the paper.

## 2 Related Work

We review prior work related to POI attribution, beginning with stay point detection, which often serves as a first step in the POI-attribution pipeline. Depending on the nature of the data collection process, it may be necessary to first extract stay points from raw GPS trajectories (Figures 1a and 1b) before a POI can be attributed to them (Figure 1c). Next, we cover relevant literature on POI attribution. Lastly, we review work on inferring check-ins from social media data, which is conceptually related to POI attribution but operates without access to trajectory data.

### Stay Point Detection.

A fundamental challenge in mobility data analysis is the identification of stay points within raw GPS trajectories. In addition to typical issues such as noisy and imprecise GPS data, there is inherent ambiguity in defining what constitutes a “stay”–specifically, how long a user must remain stationary for a stop to qualify as a stay point, and how to distinguish intentional stops (e.g., dining at a restaurant) from incidental ones (e.g., being stuck in traffic). At a high level, stay point detection methods can be categorized into clustering-, grid-, and stochastic-based approaches.

Early work in this area primarily focused on clustering-based methods, including k-means partition-based clustering [2, 4, 52] and hierarchical agglomerative clustering [12, 16]. These techniques group raw GPS coordinates into clusters representing places and may consider only spatial distance aspects [8], or incorporate both spatial and temporal dimensions [31, 52]. Grid-based or cell-based approaches have also been proposed [1, 11, 17], which divide geographic space into discrete units for analysis. Several stochastic approaches have been proposed as well [15, 36, 43], often inferring frequently-visited places as stay points from location data using probabilistic approaches such as Gaussian mixture models [32, 49] or Bayesian frameworks [30].

### Point of Interest Attribution.

A straightforward method frequently employed for POI attribution is the closest centroid approach [7]. A stay point is assigned as a visit to the POI whose centroid is closest, provided the distance between the stay point and POI centroid is below a pre-defined threshold. This method performs reasonably well in environments with large, standalone venues such as Walmart or Home Depot, where spatial ambiguity is minimal. However, its effectiveness diminishes significantly in dense urban settings where POIs are closely clustered, or where POIs vary or demonstrate irregularities in size and shape [35]. In such contexts, relying solely on proximity to the centroid often leads to incorrect attributions. These limitations have motivated the development of POI attribution methods that consider additional spatial, temporal, and semantic signals using more sophisticated probabilistic approaches.

One such approach is proposed by Nishida et al. [29], who introduce a hierarchical Bayesian model for POI attribution. After extracting stay-points from a user’s raw GPS trajectory, the model incorporates several factors to infer the most likely POI, including the dwell time, POI category information, and individual user preferences. However, a key limitation of their method is the assumption that each POI is associated with only a single category. This simplification fails to reflect the complexity of real-world locations, where POIs often span multiple categories. For example, a hotel with an in-house restaurant and spa may fall under *lodging*, *restaurant*, and *health and wellness*, while a theatre that also offers dance classes might be categorized as both *entertainment* and *education*. The inability to model such multi-category POIs limits the model’s applicability in complex urban environments. POIFormer overcomes this limitation by allowing POIs to be represented by multiple categories, enabling a more nuanced and representative attribution in diverse, multi-functional urban settings.

Suzuki et al. [41] proposed a personalized POI attribution approach under strict privacy constraints, where user visit and location data remains local to their device. As a result, POI assignment for a given user relies solely on their individual historical data without making use of aggregated information from other users’ trajectories and behaviors. Their method follows a two-step process. First, stay points are extracted using a variant of conventional methods. Second, POI attribution is formulated as an integer linear programming problem. This optimization considers spatial proximity of the stay point to POIs, dwell time at that stay point, and user-specific behavior patterns such as the user’s past visits to various POIs. The formulation of the problem as an integer linear programming, however, leads to extremely high computational costs which limits the practical efficacy of this method. POIFormer maintains computational efficiency by avoiding complexity of optimization formulation with a streamlined Transformer-based architecture that avoids complex optimization, and efficiently incorporates population-level behavioral patterns through pre-computed group-level KDEs that are readily computable from standard mobility data.

The methodology proposed by SafeGraph [35] represents the current state of the art in POI attribution and employs a comprehensive, multi-step pipeline. Beginning with raw GPS trajectories, the approach first filters out implausible GPS signals and clusters the remaining GPS signal pings to detect stay points. This is accomplished using a two-pass strategy: the first pass clusters pings within large POIs, and the second pass applies a modified DBSCAN algorithm to the remaining pings to form clusters representing potential visits. These clusters are then geospatially joined with an extensive, proprietary database of building footprint polygons that precisely delineate the physical boundaries of individual POIs. To account for any GPS inaccuracies, a buffer is added around each cluster to ensure clusters are accurately matched to the correct POI. To further refine the attribution, their approach incorporates spatial hierarchy metadata, which outlines parent-child relationships between establishments. This is particularly useful in complex structures like malls, where a store (child) is located within a larger building (parent). Next, it incorporates POI semantic context such as type of business as well as temporal information such as time of day when predicting possible POIs. Finally, a scorecard-based ranking mechanism is used to compare pairs of candidate POIs to determine which POI is the most likely for a particular stay. While highly effective, SafeGraph’s method depends heavily on their proprietary and richly annotated geospatial data layers of POI polygons and spatial hierarchy metadata – which is not available for many parts of the world. Moreover, this approach overlooks valuable behavioral signals at both the individual and population level, which can improve accuracy of POI attribution. POIFormer addresses these limitations by explicitly incorporating individual and aggregate behavioral signals, improving POI attribution accuracy (Section 4).

### Determining check-ins from social media data.

Xi et al. [45] propose a model, Bi-STDDP, that infers missing POI check-ins by integrating bi-directional spatio-temporal dependencies and users’ dynamic preferences. In essence, Bi-STDDP utilizes a user’s check-ins on social media to predict POIs that may have been visited but not explicitly recorded–i.e., “missing,” check-ins. There is no knowledge of the geographic region or location of the missing check-in; instead, it infers the most likely POI based on the other known check-ins and learned user behavior. The model effectively incorporates both global spatial and local temporal information to represent the sequential nature of user movements and their evolving behavioral patterns. Specifically, Bi-STDDP first extracts the spatial and local temporal information about POIs to glean the complex spatio-temporal dependence relationships. The target temporal pattern is then fed into a multi-layer neural network along with user and POI information to capture the dynamic user preferences. Finally, the dynamic preferences are transformed into the same space as the dependence relationships in the model. Meng et al. [21] focus on a related task – determining the purpose of a trip. The authors propose a dynamic Bayesian network model that integrates GPS trajectories, the functionality and POI popularity in trip end areas, which are learned from social media data, to infer users’ trip purposes. Trajectory sequences are then classified into one of eight categories: home, education, shopping, eating out, recreational, personal, work, and transportation.

## 3 POI Attribution with POIFormer

We now describe our methodology for assigning POIs to user stays in mobility trajectories. We formally define the problem, followed by POIFormer’s approach to leverage spatiotemporal context from user trajectories to *predict* the true POI visited during a recorded stay when multiple POI candidates are plausible.

### 3.1 Terminology

We define relevant terminology and the task of POI attribution.

#### Definition 3.1.

A *point of interest (POI)* is a specific, named location characterized by associated attributes, such as a category (e.g., ‘restaurant’ or ‘cafe’) and precise geographic coordinates. Each POI, p, is associated with a set of semantic categories, C(p), that describe its functional characteristics. We use P to denote the set of all POIs.

#### Definition 3.2.

A *stay* or *stay point* refers to a geographic region where an individual remains for a continuous duration while engaged in a contextually meaningful activity. Formally, a stay is represented as a tuple $\left(l,t^{a},t^{d},p\right)$ where l denotes the geographic location of the stay, $t^{a}$ denotes the time the individual arrived at that location for this stay, $t^{d}$ denotes the time the individual departed, and p denotes the POI visited for the stay. For brevity, we denote time tuples as $t=\left(t^{a},t^{d}\right)$ henceforth.

#### Definition 3.3.

A *trajectory* is a chronologically ordered sequence of temporally-disjoint stays recorded for a single individual. It is denoted by $S=\left\{s_{1},s_{2},\;\ldots ,s_{n}\right\}$, where each $s_{i}$ represents a stay as defined in Definition 3.2.

#### Problem Definition: POI Attribution.

In a trajectory S, stay $s_{i}=\left(l_{i},t_{i},x\right)\in S$, will need to be attributed to a location $l_{i}$.

The task of POI attribution is to identify the most probable POI p∈P that corresponds to x, the actual POI destination visited by the individual during stay $s_{i}$.

### 3.2 Methodology

In this section, we outline POIFormer, a model that can learn to predict the likelihood that a stay occurred at a POI by leveraging user trajectory and crowd-level historical patterns and stay characteristics such as time of day, category of POI, and duration. To improve the accuracy of the task of POI attribution, for a visit $s_{i}$ in an individual’s trajectory S, we not only leverage the known attributes of the stay, but also the context provided by the individual’s trajectory both before and after visit $s_{i}$ and the overall spatiotemporal priors of the stays at each POI coming from historical mobility analyses. On a high level, for a given stay point $s_{i}$ with location $l_{i}$ and a set of feasible POIs $P^{\prime }\subseteq P$, a score is computed for each POI $p\in P^{\prime }$ representing the likelihood that p is the individual’s true destination for stay $s_{i}$ given the context of the trajectory S and the known attributes of the stay $\left(l_{i},t_{i}\right)$. The POI with the highest score is considered the attributed POI for that stay.

#### 3.2.1 Capturing Individual Preferences.

For a given stay $s_{i}$ in a trajectory sequence S and the set of feasible POIs $P^{\prime }\subseteq P$ near the stay location $l_{i}$, we aim to learn a model that estimates the probability of each POI being the true destination for that stay, $Pr\left(p|s_{i},S\right)$. This captures various signals: the spatial proximity and spatiotemporal characteristics of the stay such as visit time and duration, and the user’s visit history. For clarity, we denote this going forward as Pr(p∣t,l,H) where (t,l) represents the given stay’s spatiotemporal attributes (arrival time, departure time, and location), and H represents the historical and future context surrounding the given stay $s_{i}$ within the trajectory S. Specifically, H includes the sequence of POIs visited before and after $s_{i}$, as well as their associated semantic categories. This provides the model with insight to behavioral patterns, such as whether the user typically transitions from a gym to a smoothie shop, or from work to a grocery store, which can help disambiguate between candidates in $P^{\prime }$.

We model the likelihood of a POI $p\in P^{\prime }$ being the true destination for a given stay $s_{i}$ as a function of its associated semantic categories. Each POI p has a set of associated categories, $C(p)=\left\{c_{1},c_{2},\ldots ,c_{m}\right\}$. With conditional independence assumption among the associated categories, the probability of p being the destination can be approximated as the product of individual probabilities of its constituent categories conditioned on the context of the stay and trajectory sequence. This enables several key advantages: it allows the model to scale efficiently to vast geographic regions with a diverse set of POIs; and it provides robustness in regions where data is sparse, since the estimation of individual category probabilities is more tractable and generalizable than modeling joint category distributions across all POIs. Mathematically, this is represented as:

(1) $$Pr(p|t,l,H)\approx \prod_{c\in C(p)}Pr(c|t,l,H)$$

On applying Bayes’ rule to Pr(c∣t,l,H), we arrive at:

(2) $$Pr(c|t,l,H)=\frac{Pr(t,\;l|c,\;H)\;Pr(c|H)}{Pr(t,\;l|H)}$$

Our partial goal is to obtain the category c that maximizes the term on the left. Since the term in the denominator, Pr(t,l∣H) will be the same for all categories C(p) a POI p belongs to, we omit this term. Furthermore, we make a reasonable conditional independence assumption that Pr(t,l∣c,H)≈Pr(t,l∣c) because regardless of H, the POIs visited beforehand and afterward, (1) we will only consider the set of POIs near the given location l, and (2) popular times are often similar across POIs of the same category. Thus, we can approximate Equation 2 as:

(3) Pr(c∣t,l,H)≈Pr(t,l∣c)Pr(c∣H)

Therefore, the final probability for a candidate POI p is approximately proportional to the product of the category priors given history, and the spatiotemporal likelihoods given the category, across all categories associated with that POI:

(4) $$Pr(p|t,l,H)\approx \prod_{c\in C(p)}Pr(t,l|c)\;Pr(c|H)$$

POIFormer estimates two key probabilistic components: the contextual category prior, Pr(c∣H), and spatiotemporal category likelihood, Pr(t,l∣c). The term Pr(t,l∣c) captures the distributional characteristics of each category in space and time, effectively encoding semantic patterns associated with POI categories. We train the model using a maximal likelihood approach, minimizing the cross-entropy loss between the predicted scores for the candidate POIs and ground truth label of the true destination POI.

#### 3.2.2 Capturing Crowd Preferences.

To incorporate population-level behavioral patterns Pr(t,l∣c) into POI attribution, we model crowd preferences using category-specific Kernel Density Estimators (KDEs). These KDEs capture the joint spatiotemporal distribution of visits for each POI category, reflecting how visit likelihood varies over both location and time.

Each KDE is trained offline using a large corpus of historical stay data, where the stay attributes are transformed into projected spatial coordinates, (x,y), and time (e.g., hour of day). This approach enables us to learn the fine-grained, category-dependent patterns, such as when and where visits to cafes, train stations, or clinics typically occur, effectively modeling crowd-level mobility behavior.

#### 3.2.3 Model Architecture.

**Figure 2:**
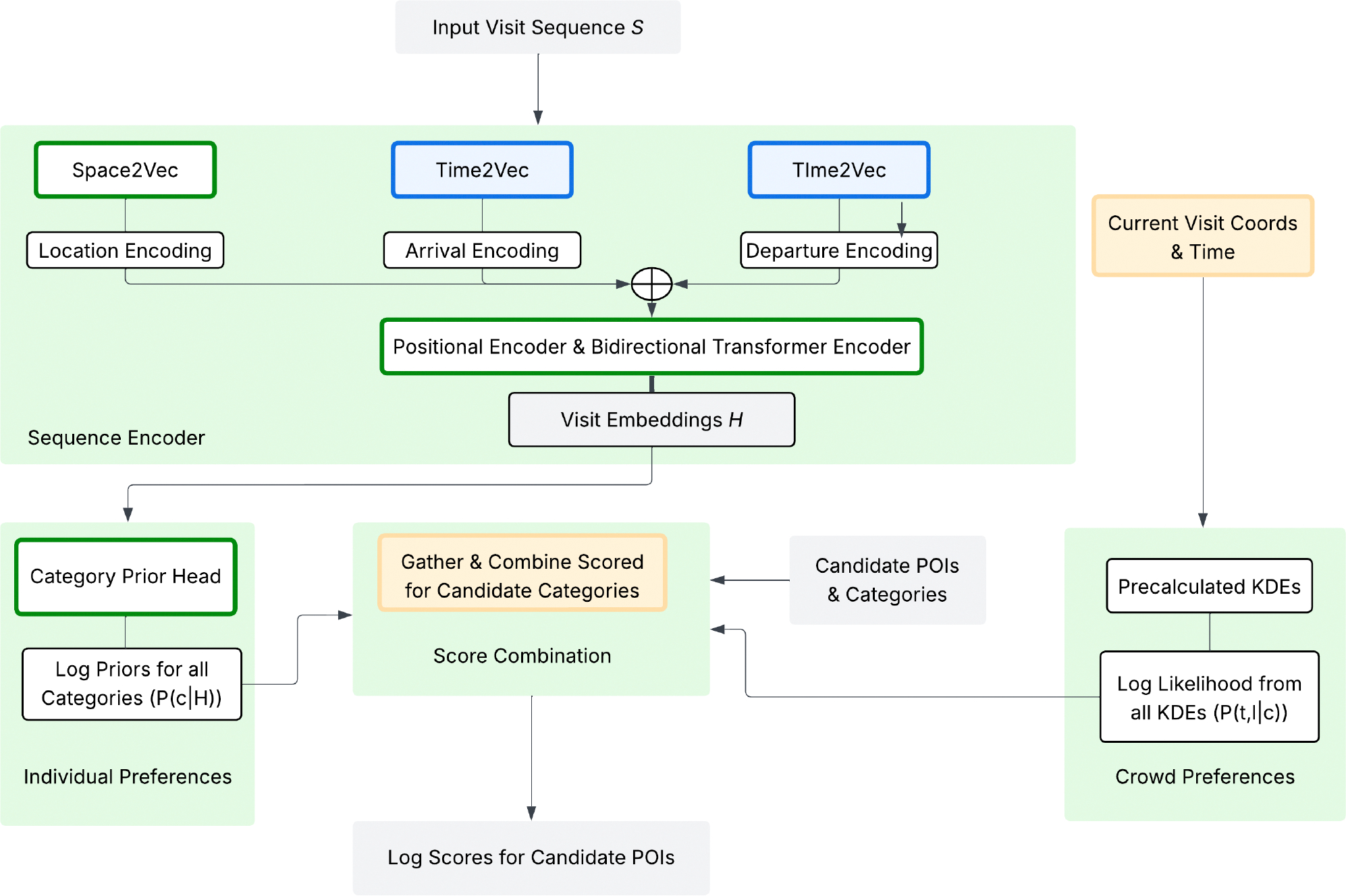
The architecture of POIFormer.

The architecture of POIFormer is illustrated in Figure 2. POIFormer operates on a given trajectory of visits S, where each stop in the sequence is observed except for a single visit of interest $s_{i}$ whose POI is unknown. The sequence encoder processes an individual’s input trajectory S to capture individual preferences, and a context extractor isolates the relevant contextual embedding corresponding to the stay of interest $s_{i}$ (Section 3.2.4). The context includes both past and future visits, capturing the time and duration of each visit. The location of the missing visit is masked, allowing the model to leverage surrounding visit patterns while inferring the most likely POI at that time. Following this, the category prior head module predicts the probability distribution over all POI categories based on the extracted context (Section 3.2.5). Meanwhile, the spatiotemporal likelihood module utilizes pre-computed kernel density estimators (KDEs) to evaluate the likelihood of the stay’s attributes given a category and the historical visit patterns (Section 3.2.6). Finally, the candidate scoring module combines the prior and likelihood information to generate a final score for each candidate POI (Section 3.2.7).

#### 3.2.4 Sequence Encoding and Context Extraction.

To capture the complex spatiotemporal dynamics in a user’s trajectory S, we first encode each stay $s_{i}=\left(l_{i},t_{i}^{a},t_{i}^{d},p_{i}\right)$ in the sequence. We use Space2Vec [20] to generate an embedding for the location coordinates $\left(x_{i},y_{i}\right)$ derived from location $l_{i}$, and Time2Vec [14] to generate separate embeddings for the arrival time $t_{i}^{a}$ and departure time $t_{i}^{d}$. If $s_{i}$ is not the stay of interest (i.e., the stay with an unknown POI), we use a learnable embedding matrix to encode $C\left(p_{i}\right)$, the categories of the stay POI. These embeddings for the location, arrival and departure times, and categories if applicable, are subsequently concatenated for each stay to form the input sequence representation.

This sequence of concatenated embeddings is then processed by a positional encoder to incorporate sequence order information. Finally, a causal transformer encoder, similar to TrajGPT’s encoder [13], is used to generate context-aware full sequence visit embeddings, H. Given H, we extract the specific hidden state $h_{i}$ corresponding to the stay of interest $s_{i}$, where $h_{i}$ represents the learned context for the stay $s_{i}$ based on the past and future trajectory.

#### 3.2.5 Category Prior Prediction.

The context embedding $h_{i}$ for stay $s_{i}$ is used to approximate logPr(c∣H)∀c, the log prior probability distribution over all possible POI categories. This is achieved using a dedicated linear layer followed by a LogSoftmax activation function:

(5) $$log\;Pr(c|H):=logSoftmax\left(Linear\left(h_{i}\right)\right)$$

This output provides a vector where each element represents the log-prior probability of visiting any POI belonging to a specific category, given the trajectory context H ending at $s_{i}$.

#### 3.2.6 Spatiotemporal Likelihood Estimation.

During model training, the relevant spatiotemporal features of the stay $s_{i}$ are extracted. To promote robustness, slight gaussian noise is added to coordinates during training. These features are then standardized using a pre-computed scaler that is fitted during KDE training, ensuring consistency across training and inference phases.

For any given POI category c, the associated pre-trained KDE is queried to compute the log-likelihood estimate, logPr(t,l∣c), of the observed spatiotemporal attributes. Specifically, we evaluate the KDE’s log probability density function at the scaled spatiotemporal representation of stay $s_{i}$, yielding logPr(t,l∣c). This likelihood provides a probabilistic measure of how well the stay’s spatiotemporal characteristics align with the aggregated behavioral patterns observed for category c, allowing the model to downweight unlikely POI candidates accordingly.

#### 3.2.7 Scoring Candidate POIs.

To compute the final score for each candidate POI $p\in P^{\prime }$, we combine the context-based prior and the spatiotemporal likelihood according to the formulation in Section 3.2.1. Since the overall probability is approximately the product $\prod_{c\in C(p)}Pr(t,l|c)\;Pr(c|H)$, we compute the log likelihood by summing the log-probabilities:

(6) $$log\;Pr(p|t,l,H)\approx \sum_{c\in C(p)}[log\;Pr(t,l|c)+log\;Pr(c|H)]$$

In other words, for each candidate POI p, its associated set of categories C(p) is retrieved. For every category c∈C(p), the log-prior probabilities, logPr(c∣H), are obtained from the output of the Category Prior Head (Section 3.2.5). Simultaneously, the Spatiotemporal Likelihood Module (Section 3.2.6) is queried to obtain the corresponding log-likelihood estimates logPr(t,l∣c). For each category c∈C(p), the log-prior and log-likelihood are summed, logPr(t,l∣c)+logPr(c∣H). Finally, these combined log-probability terms are aggregated across all categories in C(p) to produce a final score for POI p to arrive at the final score.

Appropriate masking is applied to handle padding for POIs with fewer than the maximum number of associated categories and to ignore invalid candidates based on the mask. The output is a vector of scores (logits), one for each valid candidate POI in $P^{\prime }$.

#### 3.2.8 Training and Loss.

POIFormer is trained end-to-end by minimizing the log likelihood loss between the predicted POI distribution and the ground truth label, which specifies the correct POI categories C(p). Formally, the training objective is defined as:

(7) ℒ:=-logPr(p∣t,l,H)

(8) $$\approx -\sum_{c\in C(p)}[log\;Pr(t,l|c)+log\;Pr(c|H)]$$

The training process incorporates both positive and negative examples. Optimization is carried out using a stochastic gradient-based optimizer, such as AdamW.

## 4 Evaluation

We outline our experimental setup, the publicly available datasets, evaluation metrics, and baselines used for comparison, followed by presentation of experimental results and an ablation study.

### 4.1 Experimental Setup

#### 4.1.1 Datasets.

We utilize two datasets in our experiments: NUMOSIM and Breadcrumbs. NUMOSIM [40] is a synthetic dataset designed to simulate human mobility patterns in an urban environment, specifically modeled on the city of Los Angeles, California. It is generated using deep generative models trained on real-world travel survey data from the National Household Travel Survey and empirical mobility data [9]. The dataset comprises mobility trajectories for 200,000 agents across two distinct four-week periods (one for training and one for testing), resulting in over 16 million stays. Each stay is labeled with the associated POI and corresponding POI categories. In contrast, Breadcrumbs [26] is a real-world mobility dataset collected in 2018 in Lausanne, Switzerland, specifically in an area surrounding Lake Geneva–a region with relatively low POI density compared to the highly built-up urban landscape of Los Angeles. It captures mobility data from 81 participants over a three-month period, collected via multiple smartphone sensors, including GPS, WiFI, and Bluetooth. The dataset is validated through detailed participant questionnaires, and stays are also annotated with POIs and POI categories. A summary of both datasets is given in Table 5.

#### 4.1.2 Processing.

Both datasets include pre-identified staypoints. To ensure temporal consistency, we normalize all arrival and departure timestamps by subtracting the earliest arrival time in each dataset from all other timestamps, and then convert the result into seconds. To simulate GPS uncertainty and to enhance robustness and generalizability, we perturb the true geographic coordinates in both datasets by adding zero-mean Gaussian noise. The noise standard deviation (σ) is randomly selected from the set {0.0002, 0.0001, and 0.00005}. We do not observe significant differences in results across different σ values. The NUMOSIM dataset is simulated; therefore, the coordinates of each staypoint exactly match those of its corresponding POI. We add noise to better reflect real-world conditions, where such precision is rare. The Breadcrumbs dataset is based on real-world data collected from smartphones; while some inherent noise is already present (see Table 1 for average error) and the staypoint coordinates do not necessarily coincide with the true annotated POI coordinates, we still add additional noise to increase the challenge of the task, given that the dataset covers a relatively sparse geographic region.

#### 4.1.3 Metrics.

We evaluate POI attribution performance using top-k accuracy. In other words, we measure the proportion of instances where the correct POI appears within a model’s top-k ranked predictions, offering a practical view of how well the model prioritizes relevant candidates. We report results for top-1, top-3, and top-5 accuracy to illustrate performance across varying levels of tolerance for prediction rank. Top-1 accuracy denotes the percentage of staypoints in the test set where the model’s top predicted POI matches the true POI. Top-3 and top-5 accuracies indicate the percentage of stay points in the test set for which the true POI appears among the model’s top 3 or top 5 predicted POI, respectively.

#### 4.1.4 Baselines.

We compare POIFormer with the following:

- The Closest Centroid method [7], a distance-based approach that assigns each staypoint to the nearest POI centroid.
- The probabilistic attribution model proposed by Nishida et al. [29], which models POI assignment as a probabilistic inference problem incorporating spatial and temporal features.
- The current state-of-the-art method provided by SafeGraph [35], which integrates detailed spatial layers such as building polygons and spatial hierarchy metadata with POI features to produce high-accuracy POI attribution.

#### 4.1.5 Experiments.

We conduct two sets of experiments. The first set compares POIFormer with baselines on Breadcrumbs without added noise. Since the coordinates of each staypoint in the NUMOSIM dataset exactly match those of its corresponding POI, we do not report results for NUMOSIM under noise-free conditions. The second set of experiments evaluates POIFormer against the same baselines on both Breadcrumbs and NUMOSIM with noise introduced. In each experiment, the goal is to predict (infer) the corresponding POI for each staypoint in the test set. An “experiment” in this context refers to evaluating a given method on one dataset, under one noise condition (with or without noise), and for one accuracy metric. In total, we conduct nine experiments: three experiments (Top-1, Top-3, and Top-5 accuracies) on the Breadcrumbs dataset without noise, and three experiments (Top-1, Top-3, and Top-5 accuracies) each for both datasets with added noise.

### 4.2 Experimental Results

#### 4.2.1 Experiments with added noise.

As demonstrated in Tables 2 and 3, POIFormer outperforms all baselines in five out of six noisy-data experiments across the Breadcrumbs and NUMOSIM datasets. This highlights POIFormer’s strong robustness to noise, a critical requirement for practical real-world POI attribution systems, given the inherent noise and limits to precision in GPS mobility data.Notably, POIFormer consistently delivers superior performance in the scenarios that matter most for real-world applications: crowded environments with densely clustered POIs, which are notoriously difficult to handle effectively.

We examine in more detail the single instance where SafeGraph slightly outperforms POIFormer (by 1.15% in Top-1 accuracy): the Breadcrumbs dataset under noisy conditions. All methods perform well here, which is expected given the data context–collected from 81 users in Lausanne, Switzerland, a small, low-density city of approximately 140,000 residents. Such a setting presents a limited number of POI candidates, reduced spatial ambiguity, and lower mobility diversity, all of which simplify the attribution problem and naturally lead to higher performance. In this environment, the relatively small dataset size and low number of users do not fully invoke POIFormer’s ability to leverage individual preferences and crowd-level behavioral trends–two key strengths of the model. Nonetheless, despite that, POIFormer achieves a highly competitive Top-1 accuracy of 91.69%, trailing SafeGraph by only 1.15%. This highlights the model’s versatility: even in settings where its full modeling capacity is underutilized, POIFormer delivers strong, competitive performance. At the same time, this result points to an avenue for future work: POIFormer is tailored to noisy or incomplete data, and thus its performance may be suboptimal in settings where location data are perfectly accurate. A fruitful line of investigation would therefore be to extend the model with an adaptive mechanism capable of first diagnosing the quality of the input data, since reliability of location data is not always evident a priori from the data collection process. Such a mechanism could determine whether the data are noisy, thereby warranting the use of POIFormer, or whether the data are sufficiently clean to permit simpler nearest-neighbor approaches as a more efficient alternative.

Across both datasets, POIFormer shows even more pronounced gains at Top-3 and Top-5 accuracy levels. On NUMOSIM, it surpasses the second-best method (SafeGraph) by 14.55% in Top-3 and by 15.08% in Top-5 accuracy. On Breadcrumbs, it outperforms SafeGraph by 2.96% in Top-3 accuracy and by 2.68% in Top-5 accuracy, demonstrating its consistent ability to maintain high-quality attribution across diverse and noisy environments.

These results underscore a key strength of POIFormer: it maintains robust and stable performance across varying degrees of data difficulty and noise. While SafeGraph attains near-perfect accuracy under noise-free conditions across both datasets, it exhibits notable degradation under noisy conditions and high-density settings, without recovering performance in Top-3 or Top-5 accuracy as does POIFormer. This drop reveals a critical limitation of the state-of-the-art method: while highly effective under clean conditions, it is vulnerable when exposed to the noisy data and dense settings that typify real-world urban environments.

Among all baselines, Closest Centroid exhibits the steepest performance drop under noise, underscoring its lack of robustness and its reliance on overly simplistic spatial heuristics. Its relatively strong results on the Breadcrumbs dataset are unsurprising–Lausanne’s sparse, low-density landscape makes spatial proximity alone sufficient. However, this heuristic fails to generalize to denser, more complex urban environments like NUMOSIM, where its performance degrades severely, highlighting the need for more sophisticated, noise-resilient approaches such as POIFormer.

The probabilistic model proposed by Nishida et al. [29] performs the worst overall, even trailing the simple Closest Centroid baseline. This is likely due to a key modeling limitation: the assumption that each POI belongs to a single POI category only, forcing an oversimplified representation of POI semantics that can furthermore be inaccurately inferred. Because many POIs are multi-functional, this rigid categorization constrains the model’s capacity to capture real-world visitation patterns. Notably, as shown in Table 2 and Table 3, the model performs significantly better on the Breadcrumbs dataset. We hypothesize that this is due in part to the relative simplicity of the Breadcrumbs data, which, in addition to being sparser and lower in density, predominantly contains stays annotated with a single POI category–an artifact that fortuitously aligns with the model’s single-category assumption. In contrast, this assumption breaks down in the NUMOSIM dataset, which is substantially denser and where the majority of POIs are associated with three or more categories, leading to markedly poorer performance.

Overall, these findings underscore the importance of modeling spatial and temporal dynamics while capturing both spatial contextual features of POI and behavioral features including observed individual preferences and crowd-level behavioral trends–all critical properties embodied in POIFormer. These additional features likely help to maintain robustness to noise and category ambiguity, evidenced in performance results.

#### 4.2.2 Experiments without added noise.

In the Breadcrumbs dataset with no noise, all models achieve relatively high attribution accuracy. The SafeGraph model achieves near-perfect results, with a Top-1 accuracy of 0.9997 and perfect Top-3 and Top-5 accuracies, highlighting its effectiveness when clean location signals are available. The Closest Centroid baseline also performs strongly, achieving 0.8982 Top-1 accuracy and perfect Top-3 and Top-5 accuracy. This is expected, as Lausanne’s sparse, low-density landscape allows spatial proximity alone to provide sufficient information for accurate POI inference. In contrast, Nishida et al. performs substantially worse, with a Top-1 accuracy of only 0.5271, highlighting its limitations even under ideal conditions. POIFormer also achieves strong performance in the noise-free setting. Notably, POIFormer exhibits remarkably stable performance across both clean and noisy settings, with only a minimal drop in Top-1 accuracy (from 0.9188 without noise to 0.9169 with noise), while competing methods degrade much more sharply under noise. This indicates that POIFormer learns robust representations that generalize well to realistic conditions where GPS noise is present, ensuring consistent attribution accuracy even when raw location signals are unreliable.

### 4.3 Ablation Study

To evaluate the contributions of the key components of POIFormer, we conducted an ablation study.

- POIFormer without KDE: This variant predicts the POI without leveraging the crowd-level spatiotemporal patterns modeled by the KDE, allowing us to assess the impact of population-level behavioral priors on attribution performance.
- POIFormer without learned category prior: This variant predicts the POI without utilizing the learned category prior. Since our full model estimates the likelihood of a POI p being the true destination for a given stay $s_{i}$ in part as a function of its associated semantic categories, this ablation isolates the contribution of the learned category prior to overall performance.

The ablation study is conducted on a subset of the NUMOSIM dataset comprising 100,000 stays. The results, shown in Table 4, demonstrate that POIFormer substantially outperforms both ablated variants in POI attribution across all Top-k metrics, underscoring the effectiveness of the proposed modeling approach.

Removing the KDE component leads to a notable degradation in performance: Top-1 accuracy drops by 2.31% (from 63.30% to 60.99%), Top-3 accuracy decreased by 5.13%, and Top-5 accuracy falls by 3.31%. This highlights the important role of KDEs summarizing crowd-level mobility patterns in providing fine-grained spatial and temporal likelihood estimation, which is especially valuable in complex urban settings like NUMOSIM.

The impact of removing the learned category prior is even more pronounced. Without this component, Top-1 accuracy decreases by 4.04%, and Top-3 and Top-5 accuracies drop sharply by 20.72% and 18.00%, respectively. This result confirms that modeling category-level priors is essential for disambiguating among semantically similar POIs–particularly in high-density environments where multiple POIs may be spatially proximate but serve distinct functions.

Overall, these findings demonstrate the importance of both components within POIFormer, and demonstrate that jointly modeling spatial, temporal, POI semantics, and behavioral signals is crucial for achieving robust POI attribution performance. The removal of these components results in a substantial decrease in performance, confirming their necessity in capturing the complex spatiotemporal patterns from human mobility data needed for accurate POI attribution in real-world environments.

## 5 Conclusion

We introduced POIFormer, a transformer-based model designed to leverage individual preferences and population-level behavioral trends, traditional spatiotemporal signals such as visit and dwell time and POI semantics, to improve POI attribution under noisy and sparse location data generated in dense urban environments. Comprehensive evaluations on the NUMOSIM and Breadcrumbs datasets showed that POIFormer offers substantial improvements in robustness to noise and overall attribution accuracy, particularly in complex urban environments where existing baselines degrade significantly. Moreover, the model demonstrates strong versatility, achieving state-of-the-art performance and outperforming nearly all baselines–most notably in Top-3 and Top-5 accuracy. In particular, POIFormer consistently outperforms all baselines in the scenarios that matter most for real-world applications—crowded areas with densely clustered POIs—which are notoriously challenging to handle effectively. Importantly, its versatility extends across geographies and environments: we validated it on a synthetic dataset modeled on the greater Los Angeles region as well as a real-world dataset from Switzerland, which has a very different density and POI distribution, demonstrating that POIFormer can generalize beyond a single urban context. Given its ability to model both individual and population-level behavioral context together with POI semantics, POIFormer can be readily applied to improve POI attribution in a wide range of downstream mobility analytics tasks, including personalized recommendations, urban planning, and public health exposure studies where capturing nuanced spatiotemporal and behavioral patterns is useful for improving predictive accuracy and interpretability.

## Figures and Tables

**Table 1:** Comparison of Location Positioning Methods

Method	How It Works	Avg. Accuracy Indoors / Urban	Avg. Accuracy Outdoors / Rural	Coverage and Best Use Case

**Assisted-GPS (A-GPS)** (commonly used in smartphones)	Triangulates signals from multiple satellites via radio waves by measuring signal travel time. Uses cellular assistance to speed up location fixes.	~20 m [25]	Modern devices: 7–13 m [22, 28] Older devices (such as iPhone 3G 2008): 20–30 m [48]	Global. Best for outdoor use in open areas; signal degradation likely in urban canyons or indoors due to obstruction or multipath reflections.
**Handheld GPS Devices** (e.g., Garmin GPSMAP 60Cx)	Uses satellite triangulation with advanced antennas and typically supports dual-frequency GPS for improved precision.	3–5 m [28, 48]	1.6 m (horizontal), 3.1 m (vertical) [28, 48]	High accuracy. Global coverage with good performance in most terrains.
**Wi-Fi Positioning**	Estimates location by matching nearby Wi-Fi signals to a known database of access points.	10–20 m [5, 24, 42, 44, 48]	15–40 m in suburban or semi-urban environments [24, 48]	Best in urban or dense environments; limited performance in rural or uncalibrated areas.
**Bluetooth**	Estimates location by measuring direction and strength of received signal	2–5m	up to 700m for direction estimation w/ an antenna array [39]	Susceptible to interference and multipath effects, making it less reliable in complex indoor environments.
**Cellular Network Positioning**	Determines location using timing and signal strength from nearby cell towers.	~245 m (dense urban) [23, 48]	~626 m (rural) [23, 48]	Wide coverage. Low precision; best as a fallback method.
**Hybrid (GPS, Wi-Fi, Cellular)**	Combines GPS, Wi-Fi, and cellular data to enhance positioning robustness and adaptability.	~ 4.62m in ideal conditions (i.e., sufficient Wi-Fi & cell tower signals) [10]	Accuracy degrades with weaker Wi-Fi and cellular network signals, reverting to 7–13 m [22, 28] when relying solely on GPS	Most resilient across environments; balances accuracy and coverage.

**Table 2:** Comparison of POIFormer with baseline models for POI Attribution Accuracy on the Breadcrumbs dataset with and without added noise. The best results are highlighted in bold.

Method	Accuracy w/o Added Noise			Accuracy with Added Noise		
	Top-1	Top-3	Top-5	Top-1	Top-3	Top-5

Closest centroid [7]	0.8982	**1.000**	**1.000**	0.6947	0.8696	0.9032
Nishida et al. [29]	0.5271	0.6749	0.6995	0.4926	0.6305	0.6355
SafeGraph [35]	**0.9997**	**1.000**	**1.000**	**0.9276**	0.9517	0.9655
**POIFormer**	0.9188	0.9924	**1.000**	0.9169	**0.9799**	**0.9914**

**Table 3:** Comparison of POIFormer with baseline models for POI Attribution Accuracy on the NUMOSIM dataset with noise. POIFormer outperforms all baselines, especially in crowded, densely clustered POI settings—key real-world scenarios that are especially challenging. Best results are highlighted in bold.

Method	Top-1 Accuracy	Top-3 Accuracy	Top-5 Accuracy

Closest centroid [7]	0.2649	0.5911	0.7570
Nishida et al. [29]	0.1002	0.1774	0.2014
SafeGraph [35]	0.7023	0.7938	0.8388
**POIFormer**	**0.7223**	**0.9093**	**0.9653**

**Table 4:** Ablation study of POIFormer components for POI Attribution Accuracy.

Method	Top-1 Accuracy	Top-3 Accuracy	Top-5 Accuracy

POIFormer w/o KDE	0.6099	0.8453	0.9183
POIFormer w/o learned category prior	0.5926	0.6894	0.7714
**POIFormer**	**0.6330**	**0.8966**	**0.9514**

**Table 5:** Comparison of Breadcrumbs and NUMOSIM Datasets

Attribute	Breadcrumbs	NUMOSIM

**Dataset Type**	Real-world mobility dataset	Synthetic mobility dataset with injected anomalies
**Data Collection Method**	Multi-sensor smartphone data (GPS, GSM, Wi-Fi, Bluetooth) from 81 participants	Simulated data using deep learning models trained on real mobility data
**Temporal Coverage**	12 weeks between March and June 2018	Simulated over multiple weeks
**Spatial Coverage**	Lausanne, Switzerland	Large-scale simulation of Los Angeles, CA
**Number of Users/Agents**	81 individuals	200,000 simulated agents
**Data Modalities**	GPS, GSM, Wi-Fi, Bluetooth, contact records, calendar events, lifestyle information	GPS trajectories, stay points, check-ins, trip durations, trip purposes
**Annotations**	Semantic labels for points of interest (e.g., “home”, “work”), demographic attributes, social relationship labels. Stays are annotated with ground truth POIs, POI category confirmed by users.	Annotated anomalies (e.g., centralized manipulation, infectious disease spread), demographic and social attributes. Stays are annotated with POIs and POI categories.

## CCS Concepts

- • Information systems → Location based services; • Computing methodologies → Neural networks.
